# p73 expression is associated with cellular chemosensitivity in human non-small cell lung cancer cell lines

**DOI:** 10.3892/ol.2012.1035

**Published:** 2012-11-19

**Authors:** KAISHAN LIU, XIAOMEI ZHUANG, ZHUOYING MAI

**Affiliations:** Department of Pathology, School of Medicine, Jinan University, Guangzhou, Guangdong 510632, P.R. China

**Keywords:** p73 gene, methylation, chemosensitivity, non-small cell lung cancer

## Abstract

p73 is a member of the p53 tumor suppressor protein family and induces apoptosis in tumor cells that lack functional p53. It has been demonstrated that methylation of CpG islands in the promoter and exon 1 region may result in silencing of the p73 gene. The aim of this study was to investigate the correlation between p73 gene expression and chemosensitivity in non-small cell lung cancer (NSCLC) cell lines. The expression of the p73 transcript in six NSCLC cell lines was investigated by reverse transcription-polymerase chain reaction (RT-PCR). The methylation status in these cell lines was determined by methylation-specific PCR (MSP) analysis. An *in vitro* demethylation assay was conducted using the DNA methyltransferase inhibitor 5-aza-2-deoxycytidine (5-aza-dC). Restored expression of p73 in the human lung squamous cell carcinoma cell line C57, both at the mRNA and protein level, was investigated by RT-PCR and immunohistochemistry, respectively. A colony formation assay was used to measure the surviving fraction of the C57 cell line. Transcript silencing of the p73 gene in the six NSCLC cell lines was observed and related to aberrant methylation. The expression of the p73 transcript and protein in the C57 cell line was restored by 5-aza-dC. The surviving fraction for colony formation in C57 cells pre-treated with 5-aza-dC was 0.059±0.006, which was significantly different from that of the control group (0.12±0.008; P<0.05). Our data demonstrated a significant correlation between expression of p73 and cellular chemosensitivity in NSCLC.

## Introduction

Lung cancer is the most common form of cancer worldwide. Non-small cell lung cancer (NSCLC) accounts for ∼80% of all lung cancer cases and is typically associated with frequent development of resistance towards chemotherapy. Deficiency in apoptosis is considered to be a major cause of the therapeutic resistance of NSCLC, as the chemotherapeutic drug cisplatin is one of the most frequently used agents in NSCLC treatment, and its cytotoxic effects are speculated to be due to the DNA damage and apoptosis it induces in cells (1,2).

Apoptosis is activated and inactivated by a variety of genes. Deregulation of genes involved in the activation or execution of the apoptotic process may lead to chemoresistance in cells. p53-dependent apoptosis is a significant mechanism whereby DNA-damaging cisplatin exerts its biological effects (3). However, apoptosis is capable of occurring in p53-deficient cells. It has been demonstrated that p73 functionally replaces p53 in adriamycin-treated, p53-deficient breast cancer cells (4,5). The data suggest that p53-independent pathways exist and these are involved in the cellular response to anti-cancer drugs.

p73 shares a marked homology in DNA sequence and protein structure with p53. As with p53, p73 is a tumor suppressor gene. The p73 gene maps to chromosome 1p36 and encodes two different proteins that are expressed under the control of two independent promoters, and that have opposite functions: The transcriptionally active full-length TAp73 and the NH_2_-terminally truncated dominant-negative ΔNp73. TAp73 has been demonstrated to be involved in cellular responses to DNA damage induced by chemotherapeutic agents. The promoter was capable of inhibiting cell growth in a p53-like manner by inducing apoptosis. However, in contrast with p53, mutation of p73 has rarely been found in the majority of human cancers (6,7). The transcription of p73 is regulated by the promoter and exon 1, which is rich in CpG dinucleotides. Methylation of the cytosine residues at the CpG dinucleotides within this region plays a critical role in inactivating gene expression (8,9).

As p53 function is often impaired in NSCLC, it would be valuable to understand whether p73 in NSCLC is capable of compensating for the impaired p53 function and thus triggering p53-independent apoptosis of cancer cells in response to chemotherapy. Therefore, the aim of this study was to investigate the correlation between p73 gene expression and chemosensitivity in NSCLC cell lines.

## Materials and methods

### Cell lines and cultures, and 5-aza-2-deoxycytidine (5-aza-dC) treatment

Two cell lines derived from squamous cell carcinomas of the lung (SK-MES-1 and C57), three cell lines derived from adenocarcinomas of the lung (A549, GLC and P15) and one cell line derived from a large cell lung carcinoma (NCI-H460) were obtained from the American Type Culture Collection (Manassas, VA, USA) or the Animal Experiment Center (Sun Yat-sen University, Guangzhou, China). The breast cancer cell line MCF7 was also included to validate the reverse transcription-polymerase chain reaction (RT-PCR) approach to p73 expression. The five cell lines (C57, GLC, P15, NCI-H460 and A549) were maintained in RPMI-1640 medium supplemented with 10% fetal bovine serum (FBS) in 5% CO2 at 37°C. SK-MES-1 cells were grown in α-Minimum Essential medium supplemented with 10% FBS and antibiotics. Cells were harvested with 0.25% trypsin. The human lung squamous cell carcinoma cell line C57 was exposed to different concentrations (2, 5 and 10 *μ*mol/l) of the demethylation agent 5-aza-dC (Sigma, St. Louis, MO, USA) for 72 h, to assess restoration of p73 gene expression.

The study was approved by the Ethics Committee of the School of Medicine, Jinan University, Guangzhou, Guangdong, China.

### Total RNA extraction and RT-PCR

Total RNA was extracted from cells using TRIzol reagent (Omega Bio-Tek, Norcross, GA, USA). After purification, RNA was dissolved in diethylpyrocarbonate (DEPC)-treated distilled water. The cDNA was synthesized with random hexamer primers and stored at −20°C until use. Total RNA (1.0 *μ*g) was reverse-transcribed by AMV Reverse Transcriptase (Takara Biotechnology; Dalian, China), from which the cDNA was obtained for PCR amplification. The PCR reaction was performed using primers spanning exons 5 and 6 of the p73 gene without amplification of genomic sequences (p73–715 forward, 5′-ACTTCAACGAAGGACAGTCTGCT and p73–856 reverse, 5′-AATTCCGTCCCCACCTGTG) (10). RT-PCR was performed for 1 cycle at 94°C for 2 min, followed by 30 cycles at 94°C for 30 sec, 63°C for 30 sec and 72°C for 1 min. The length of the PCR product for the p73 transcript was 142 bp. β-actin gene was selected as an endogenous reference.

### DNA extraction, sodium bisulfite modification of DNA and methylation-specific PCR (MSP)

Genomic DNA was prepared from the cultured cells. DNA was extracted by OB Protease and Tissue Isolation Reagent (Omega Bio-Tek) according to the manufacturer’s instructions. The methylation status within the CpG island of the p73 gene in exon 1 (sequence 110-42 bp relative to translation start, GenBank Accession number Y11416) was determined by MSP using bisulfite-modified DNA (11). Bisulfite modification was conducted using the EZ DNA Methylation-Gold kit (Zymo Research Co.; Beijing, China). Following this reaction, all unmethylated cytosines were deaminated and converted to uracil, while methylated cytosines remained unchanged. Following bisulfite conversion, methylated and unmethylated genomic regions were distinguished by PCR using each sequence-specific pair of primers. Primer sequences for methylated and unmethylated alleles of p73 are presented in Table I. Unmethylated and methylated fragments were amplified under the following PCR reactions: 35 cycles of 30 sec at 94°C, 30 sec at 59°C and 30 sec at 72°C for the unmethylated products; whereas methylated products were amplified in 40 cycles of 30 sec at 94°C, 30 sec at 60°C and 30 sec at 72°C. In both cases an initial denaturation of 7 min at 94°C and a final extension of 5 min at 72°C were conducted. PCR products were electrophoresed on 3% agarose gel and visualized by ethidium bromide staining. Human placental DNA treated *in vitro* with Sss I methylase (New England Biolabs, Inc.; Ipswich, MA, USA) served as a positive control for the methylated reaction. Control reactions without DNA were performed alongside each PCR.

### Immunohistochemistry

After washing with phosphate-buffered saline (PBS), slides were incubated in diluted primary antibody at room temperature for 2 h. The p73 (E-4) antibody used was a mouse monoclonal antibody (Santa Cruz Biotechnology, Inc.; Santa Cruz, CA, USA) raised against amino acids 1–80 and mapping at the N-terminus of p73 of human origin, which recognizes all human TAp73 isoforms. The mouse anti-human p73 monoclonal antibody was used at a 1/50 dilution for a final concentration of 4.0 mg/l. Slides were subsequently incubated in 10% normal horse serum for 20 min. Then, the sections were added to poly-horse, rabbit-peroxidase-anti mouse/rabbit IgG (Maixin, Biotechnology Development Co., Ltd; Fuzhou, China). After 30 min, the sections were rinsed with PBS. Development of the slides was performed using 3, 3′-diaminobenzidine (DAB) solution. Hematoxylin was used as the nuclear counterstain. Immunoreactivity was confirmed as positive for the p73 nuclear identification. In all runs, negative controls were included and PBS was substituted for the primary antibody; staining was not observed.

### Clonogenic survival assay

Following treatment with 5 *μ*mol/l 5-aza-dC for 72 h, the exponentially growing cells were trypsinized into a single cell suspension. Cell viability was assessed by trypan blue dye exclusion. Viable cells (5x10^2^) were plated in 60 mm Petri dishes and exposed to the chemotherapeutic drug cisplatin (1.25 *μ*mol/l) for 48 h. Following exposure, cells were incubated at 37°C in 5% CO_2_ to facilitate colony formation. Following growth for 10–14 days, the colonies were fixed with methanol and stained with Giemsa dye (Huamei Biotechnology Co., Ltd., Beijing, China). Colonies exhibiting a minimum of 50 viable cells were counted. Colony plating efficiency was calculated to be the number of viable plated cells, and was expressed as a percentage of inoculated cells.

### Statistical analysis

The results were expressed as the mean ± standard deviation. MS Excel 7.0 computer software was used to perform statistical analyses. A two-tailed Student’s t-test was used for statistical comparisons. P<0.05 was considered to indicate a statistically significant difference.

## Results

### Expression of p73 in human lung carcinoma cell line

**Figure 1.**
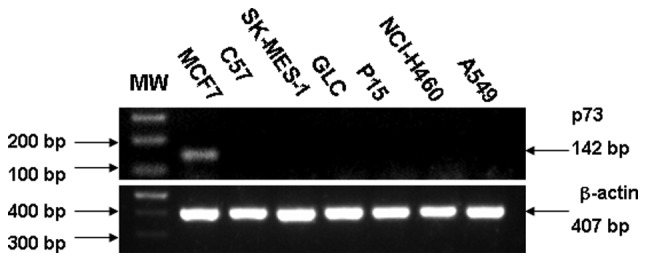
Expression of p73 in the six lung cancer cell lines at RNA level by reverse transcription-polymerase chain reaction (RT-PCR). β-actin was expressed as an endogenous reference. The breast cancer cell line MCF7 was included as expression control. MW, molecular weight marker (100 bp).

Expression of p73 at the RNA level is illustrated in Fig. 1. A sample was considered negative when it exhibited positive staining for β-actin and negative staining for p73. SK-MES-1 and C57 derived from human lung squamous cell carcinoma did not express any p73 mRNA. GLC, P15, A549 and NCI-H460, derived from adenocarcinoma of lung and large cell lung carcinoma respectively, failed to express p73 at the RNA level.

### p73 methylation status

**Figure 2.**
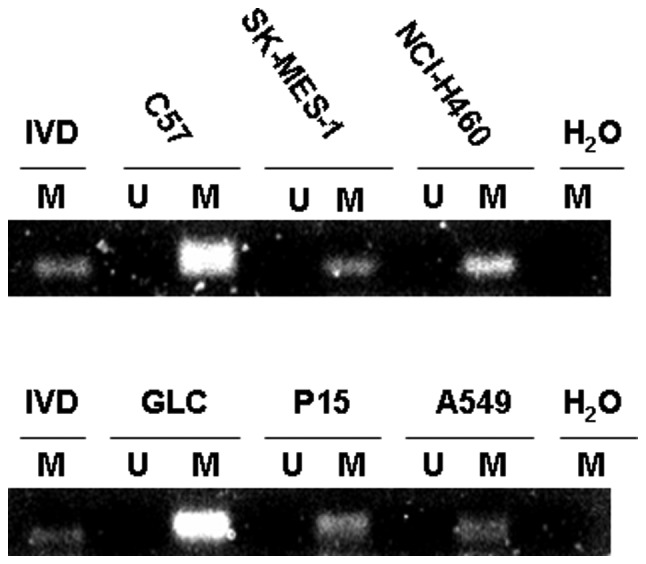
Methylation-specific polymerase chain reaction (PCR) analysis of the six non-small cell lung cancer cell lines. M, methylated fragments; U, unmethylated fragments; IVD, *in vitro* methylated placental DNA by SssI methylase as a positive control for methylation.

To determine whether aberrant methylation of p73 occurred in human NSCLC, we first investigated six NSCLC cell lines. By MSP, all six cell lines including C57, SK-MES-1, GLC, P15, A549 and NCI-H460 demonstrated evidence of methylation in exon 1 (Fig. 2). The observed methylation patterns correlated with transcriptional silencing of the p73 gene. The p73 transcript was undetectable in the six methylated cell lines by RT-PCR (Fig. 1).

### Restoration of p73 gene expression

**Figure 3.**
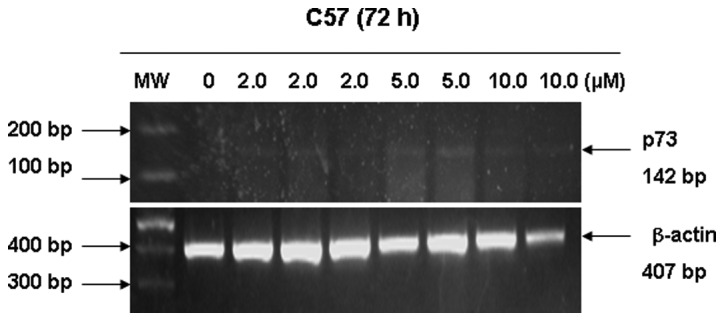
5-aza-dC restores the expression of p73 at mRNA level in cell line C57 by reverse transcription-polymerase chain reaction (RT-PCR). PCR products of p73 (142 bp) were detected and β-actin gene (407 bp) was used as an internal control. 5-aza-dC was applied at 2.0, 5.0 and 10.0 *μ*mol/l for 72 h.

**Figure 4.**
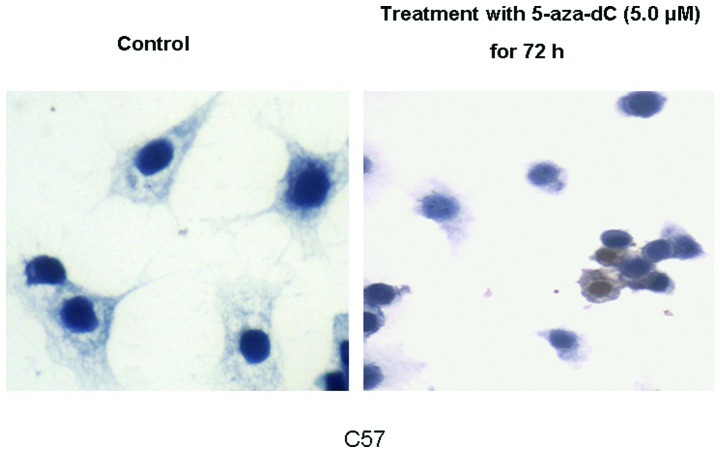
5-aza-dC restores the expression of p73 in cell line C57 at the protein level by immunohistochemistry. The concentration of 5-aza-dC was 5 *μ*mol/l for 72 h. Original magnification, x400. Brown color of cell nuclei was identified as positive p73 staining.

Human lung squamous cell carcinoma cell line C57 was exposed to different concentrations of 5-aza-dC to restore expression of p73 transcripts. As demonstrated in Fig. 3, 5-aza-dC induced re-expression of the p73 gene at the mRNA level by RT-PCR. p73 mRNA level was abundant at 5 *μ*mol/l 5-aza-dC, and was weakly detected at 2 and 10 *μ*mol/l 5-aza-dC. Immunohistochemistry revealed that low and high expression of the p73 protein was restored in the C57 cell line following exposure to 5 *μ*mol/l 5-aza-dC (Fig. 4).

### Demethylation of p73 in the C57 cell line increases chemotherapeutic drug-(cisplatin-) induced cell death

**Figure 5.**
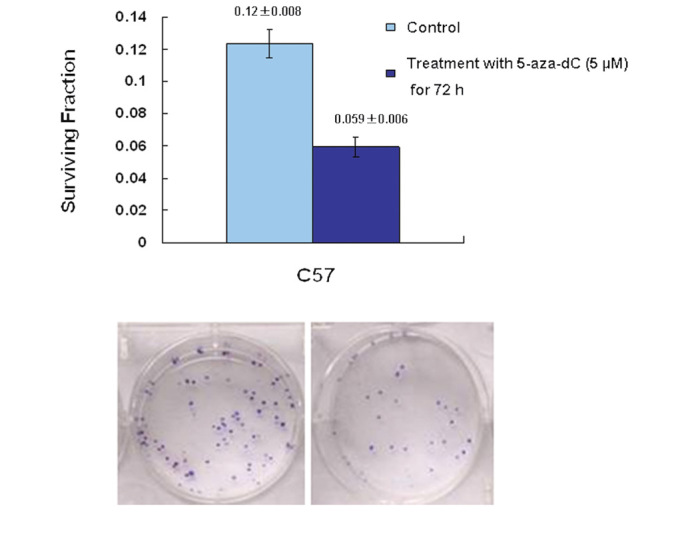
After treatment of 5-aza-dC for 72 h, C57 cells were exposed to chemotherapeutic drug cisplatin for 48 h and colony formation was measured by clonogenic cell survival 10–14 days later (data are the mean of triplicate experiments, P<0.05).

In order to explore how modulation of p73 status in the C57 cell line influenced chemosensitivity, C57 cells were treated with 5-aza-dC and subsequently assayed for clonogenic survival following treatment with 1.25 *μ*mol/l cisplatin. The results indicated that upregulation of the level of p73 in cells, induced by 5-aza-dC, significantly increased the chemosensitivity of C57 cells; the p73 promoter was demethylated and p73 gene expression was restored. The survival fraction for colony formation in cells pre-treated with 5-aza-dC was 0.059±0.006. This was significantly different from that of the control group (0.12±0.008; P<0.05) (Fig. 5).

## Discussion

Although chemotherapeutic agents including cisplatin are widely used in the treatment of lung cancer, chemoresistance remains a significant problem. The cytotoxic effect of cisplatin is attributed to the formation of bulky DNA adducts, causing DNA damage and finally inducing cancer cell apoptosis. Therefore, the cellular apoptosis pathway may play a key role in chemosensitivity (12).

p73 is a member of the p53 tumor suppressor protein family. As with p53, p73 elicits cancer cell apoptosis in response to DNA damage caused by cisplatin-based chemotherapy. Also, p73 is capable of inducing apoptosis in tumor cells that lack functional p53. Combination of p73 gene therapy and chemotherapy has been demonstrated to be more effective compared with either of these agents alone in a clinical trial for patients with NSCLC (13). However, epigenetic silencing of the p73 gene via promoter and exon 1 hypermethylation has been demonstrated to be a common event in types of lymphoma, brain tumor and cervical cancer (14–16). In the present study, the epigenetic modification of p73 via CpG island methylation represented a critical mechanism for inactivation of this gene in six NSCLC cell lines (Fig. 2). Hypermethylation was significantly correlated with loss of p73 expression in these cell lines (Fig. 1). *In vitro* demethylation assay by the DNA methyltransferase inhibitor, 5-aza-dC was successful in restoring p73 mRNA (Fig. 3) and protein (Fig. 4) expression levels in the human lung squamous cell carcinoma cell line C57. Furthermore, following restoration of p73 expression by 5-aza-dC treatment, chemotherapeutic agent cisplatin-induced cell death was increased in the C57 cell line (Fig. 5). Although 5-aza-dC was not a p73-specific demethylation agent, the change in p73 expression induced by 5-aza-dC influenced the cisplatin-based chemosensitivity in the C57 cell line.

At present, a large number of genes have been investigated for their methylation status in lung cancer patients. Genes that have been intensively studied in primary NSCLC include p16 (cyclin-dependent kinase inhibitor 2A), RASSF1A (Ras association domain family protein 1A), APC (adenomatous polyposis coli), RARβ-2 (retinoic acid receptor β), DAPK (death-associated protein kinase) and MGMT (O6-methylguanine DNA methyltransferase). While p16 and RASSF1A are involved in cell cycle regulation, APC inhibits β-catenin, RARβ-2 is involved in growth regulation, DAPK plays a role in the regulation of apoptosis and MGMT functions in DNA repair. Overall, detecting the methylation status in these genes has the potential to aid prediction of disease recurrence after surgery, monitoring of the responses to therapy and early detection of NSCLC. In addition, it may also be a therapeutic target (17). Restoration of p73 expression is highly desirable, as p73 has been suggested to have a role in determining cellular chemosensitivity.

In conclusion, the present study demonstrated the significant correlation between expression of p73 and cellular chemosensitivity in NSCLC that has been treated with cisplatin. Therefore, p73 may play an important role in regulating the cellular response of NSCLC to chemotherapy. The underlying mechanism of p73 signaling in the apoptosis pathway during chemotherapy requires further investigation. Whether the methylation status of the p73 gene is a potential predictive marker in evaluating the treatment regimens of NSCLC patients is a subject that requires validation in a prospective study with a large group of NSCLC patients.

## Figures and Tables

**Table I.** Primer sequences for methylated and unmethylated alleles of p73.

p73 allele	Primer pairs
Methylated	5′-Primer 5′-GGACGTAGCGAAATCGGGGTTC-3′
	3′-Primer 5′-ACCCCGAACATCGACGTCCG-3′
Unmethylated	5′-Primer 5′-AGGGGATGTAGTGAAATTGGGGTTT-3′
	3′-Primer 5′-CCATCACAACCCCAAACATCA-3′

## Abbreviations:

NSCLC: non-small cell lung cancer;
5-aza-dC: 5-aza-2-deoxycytidine;
MSP: methylation-specific PCR
